# Protein arginine methyltransferase 7-mediated arginine mono-methylation stabilizes SRY-box transcription factor 9 to promote non-small cell lung cancer progression

**DOI:** 10.1186/s43556-025-00378-0

**Published:** 2025-12-22

**Authors:** Lin Zhang, Jingyi Xiang, Yali Feng, Gufang Shen, Xu Huang, Tianshu Fang, Yunjia Zhu, Hong Ren, Chungang Liu

**Affiliations:** 1 Key Laboratory of Molecular Biology for Infectious Diseases (Ministry of Education), Institute for Viral Hepatitis, Department of Infectious Diseases, The Second Affiliated Hospital, Chongqing Medical University, Chongqing, 400010 People’s Republic of China; 2 Mental Health Center of Jiulongpo District, Chongqing, 401329 People’s Republic of China; 3 Department of Epidemiology, The First Affiliated Hospital of Shandong First Medical University & Shandong Provincial Qianfoshan Hospital, Jinan, Shandong Province 250000 People’s Republic of China; 4 Department of Pathology, The Second Affiliated Hospital of Chongqing Medical University, Chongqing, 400010 People’s Republic of China

**Keywords:** Non-small cell lung cancer, Protein arginine methyltransferase 7, SRY-Box transcription factor 9, Arginine mono-methylation

## Abstract

Non-small cell lung cancer (NSCLC) remains a leading cause of cancer-related mortality worldwide, underscoring the need for deeper insights into its molecular mechanisms. The SRY-box transcription factor 9 (SOX9) is a well-characterized oncoprotein critical for the pathogenesis and progression of multiple cancers, including NSCLC. However, the post-translational mechanisms governing SOX9 stability, particularly those mediated by arginine methyltransferases, are poorly defined. In this study, we identify protein arginine methyltransferase 7 (PRMT7) as a novel regulator of SOX9. We demonstrate that PRMT7 directly interacts with SOX9 and enhances its stability in a methyltransferase-dependent manner. Mechanistic investigations reveal that PRMT7 catalyzes mono-methylation of SOX9 at the arginine 160 (R160) residue, which in turn antagonizes SOX9 ubiquitination and subsequent proteasomal degradation mediated by the E3 ligases F-Box and WD repeat domain containing 7 (FBXW7) and kelch like ECH associated protein 1 (KEAP1). Functional assays revealed that PRMT7 promotes NSCLC cell proliferation in vitro and tumorigenesis in vivo in a SOX9-dependent manner. Consistent with these findings, clinical analysis revealed significant co-upregulation of both PRMT7 and SOX9 in NSCLC specimens relative to adjacent normal tissues, with elevated levels of either protein correlating with diminished patient survival. Collectively, our findings establish PRMT7 as a key regulator of SOX9 stability and function, thereby highlighting the PRMT7-SOX9 axis as a promising therapeutic target in NSCLC.

**Supplementary Information:**

The online version contains supplementary material available at 10.1186/s43556-025-00378-0.

## Introduction

Lung cancer is the most common cause of cancer-related mortality worldwide. The vast majority of cases (approximately 85%) are classified as non-small-cell lung cancer (NSCLC) [1–4]. Despite advances in diagnosis and treatment over the previous decades, the incidence and mortality rates of NSCLC remain high and are still increasing [5–7]. Therefore, a deeper understanding of the molecular mechanisms underlying NSCLC progression could have important clinical implications.

The SRY-Box transcription factor 9 (SOX9) plays a pivotal role in embryonic development and tumorigenesis [8–10]. In NSCLC, high SOX9 expression correlates with advanced histological stage and poor prognosis [11]. The expression and activity of SOX9 are tightly regulated by transcriptional and post-translational modifications (PTMs) [12]. Previously, we and others have demonstrated that SOX9 protein is degraded by KEAP1 or FBXW7 via the ubiquitin–proteasome system [13–15]. Emerging evidence indicates that arginine and lysine methylation can regulate SOX9 stability by modulating its ubiquitination [16–18]. For instance, lysine demethylase 3 A stabilizes SOX9 by demethylating lysine 68 and attenuating its ubiquitination [17]. Conversely, asymmetric dimethylarginine promotes SOX9 degradation by impeding USP7-mediated deubiquitination [16]. However, whether arginine methylation directly regulates SOX9 protein stability in NSCLC remains unknown.

Protein arginine methylation, a process catalyzed by protein arginine methyltransferases (PRMTs), is a pivotal post-translational modification that regulates diverse cellular processes including subcellular localization and protein stability [19–22]. Among the three catalytic types of PRMTs, PRMT7 is unique as the only type III enzyme that exclusively catalyzes arginine mono-methylation [23–26]. PRMT7 can function as either a tumor suppressor or an oncogene in a context-dependent manner, primarily by methylating histone (e.g., H2A, H4) and non-histone substrates [19, 27–35]. For instance, PRMT7 methylates PTEN to suppress the PI3K/AKT pathway in gastric cancer [35], and NUDT21 to counteract enzalutamide resistance in prostate cancer [34]. Paradoxically, in breast cancer, PRMT7 methylates eukaryotic translation initiation factor 2α (eIF2α) to promote drug resistance [30], whereas in renal-cell carcinoma, it methylates β-catenin to enhance its stability and promote proliferation [33]. Despite these advances, the specific substrates and mechanistic roles of PRMT7 in non-small cell lung cancer (NSCLC) remain poorly defined.

Here, we identified PRMT7 as a novel methyltransferase that mono-methylates SOX9 at the R160 residue. This modification consequently antagonizes ubiquitination and degradation induced by KEAP1 or FBXW7, thereby stabilizing the SOX9 protein. Furthermore, PRMT7 promoted NSCLC cell proliferation in vitro and tumorigenesis in vivo in a SOX9-dependent manner. Analysis of clinical specimens revealed that high co-expression of PRMT7 and SOX9 was correlated with poor prognosis in NSCLC patients. Collectively, our findings designate the PRMT7-SOX9 axis as a potential therapeutic target for NSCLC.

## Results

### PRMT7 acts as a positive regulator of the SOX9 protein abundance in a methyltransferase activity-dependent manner

**Fig. 1:**
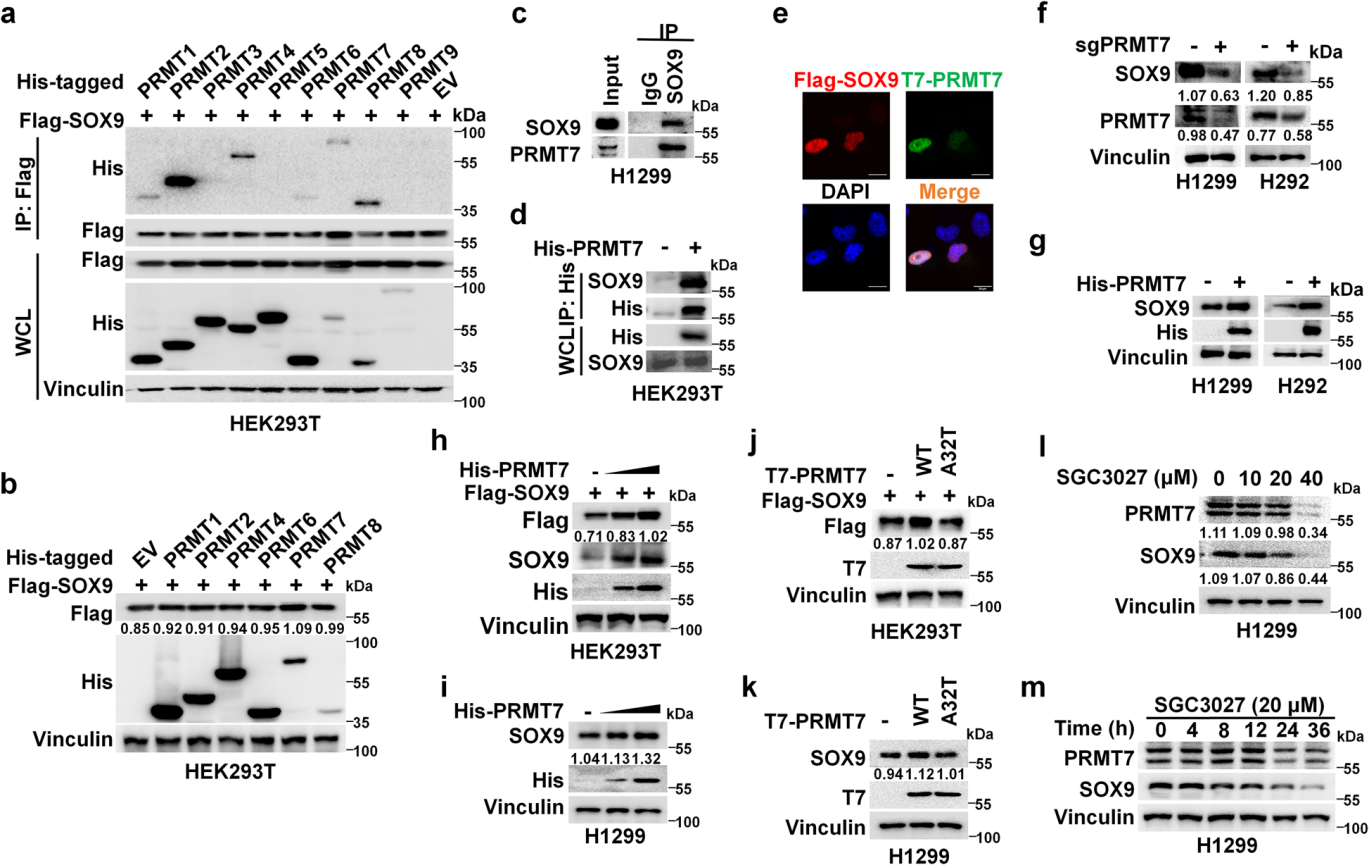
PRMT7 acted as a positive regulator of the SOX9 protein abundance in a methyltransferase activity-dependent manner. **a** Immunoblotting (IB) analysis of whole-cell lysates (WCL) and immunoprecipitation (IP) from HEK293T cells transfected with empty vector (EV) or various His-tagged PRMT constructs and vectors encoding Flag-tagged SOX9 (Flag-SOX9). **b** IB analysis of SOX9 protein levels in HEK293T cells co-transfected with Flag-SOX9 together with or without His-tagged PRMT constructs. **c** PRMT7 interacts with SOX9 in vivo. H1299 cells were immunoprecipitated with anti-SOX9 antibody and then analyzed by IB. Input is 5% of the total lysates used in IP. **d** Co-IP of His-tagged PRMT7 and endogenous SOX9 in HEK293T cells. Cells were transfected with either empty vector or His-PRMT7 plasmid. Cell lysates were immunoprecipitated with anti-His antibody, followed by IB with anti-SOX9 antibody. **e** Flag-SOX9 was co-expressed with T7-PRMT7 in H1299 cells. The cellular localization of SOX9 (Red) and PRMT7 (Green) was examined by immunofluorescence (IF) staining (nuclei were stained with DAPI; blue). Scale bar, 20 μm. **f** IB analysis of SOX9 expression level in H1299 and H292 cells with PRMT7 knockout. Parental H1299 or H292 cells were used as the control. **g** IB analysis of endogenous SOX9 protein levels in H1299 or H292 cells transfected with His-PRMT7. Cells were harvested after 36 h post-transfection. **h** HEK293T cells were transfected with indicated concentration (0.5 μg, 1 μg) of His-PRMT7 plasmid and Flag-SOX9 plasmid and then harvested for IB analysis. **i** H1299 cells were transfected with indicated concentration (0.5 μg, 1 μg) of His-PRMT7 plasmid and then harvested for IB analysis. **j**, **k** Immunoblot (IB) analysis of the SOX9 protein levels in HEK293T (**j**) or H1299 (**k**) cells transfected with indicated plasmids. **l**, **m** IB analysis of endogenous SOX9 protein levels in H1299 cells treated with increasing concentrations of SGC3027 for 12 h (**l**), or 20 μM SGC3027 for different time (**m**)

To identify the PRMTs that regulate SOX9 protein abundance, we overexpressed individual PRMTs in human embryonic kidney 293 T (HEK293T) cells and monitored SOX9 levels. Our results indicated that only PRMT7, not other the tested PRMTs, specifically bound to and significantly increased the protein abundance of SOX9 (Fig. 1a-b and Fig. S1a). The PRMT7-SOX9 interaction in the nucleus was further confirmed by endogenous and semi-endogenous co-immunoprecipitation along with immunofluorescence staining assays (Fig. 1c-e). Knockout of PRMT7 led to a significant decrease in the SOX9 protein abundance (Fig. 1f and Fig. S1b). Conversely, overexpression of PRMT7 significantly increased the exogenous and endogenous protein abundance of SOX9 in a dose-dependent manner (Fig. 1g-i and Fig. S1c), without affecting the mRNA levels (Fig. S1d). Furthermore, the lung carcinoma datasets from cBioPortal showed that SOX9 mRNA levels did not correlate with PRMT7 mRNA levels (Fig. S1e). These results suggest that SOX9 protein levels are regulated in a post-translational manner, partially by PRMT7.

Given that PRMT7 acts as an arginine methyltransferase [35], we postulated that PRMT7’s methyltransferases activities are critical for maintaining SOX9 protein abundance. We found that overexpression of the wild type (WT), not the catalytically inactive PRMT7 mutant [A32T (Ala32 to Thr)], led to an increase in the SOX9 protein abundance (Fig. 1j-k). Consistent with the genetic ablation of PRMT7, pharmacological inhibition of PRMT7 by the specific inhibitor SGC3027 [31] led to a decrease in SOX9 protein abundance in a dose- and time-dependent manner (Fig. 1l-m, Fig. S1f). Collectively, these results indicated that PRMT7 had a critical role in increasing SOX9 protein abundance in a methyltransferase activity-dependent manner.

### PRMT7 enhances SOX9 protein stability by inhibits its ubiquitination and degradation

**Fig. 2:**
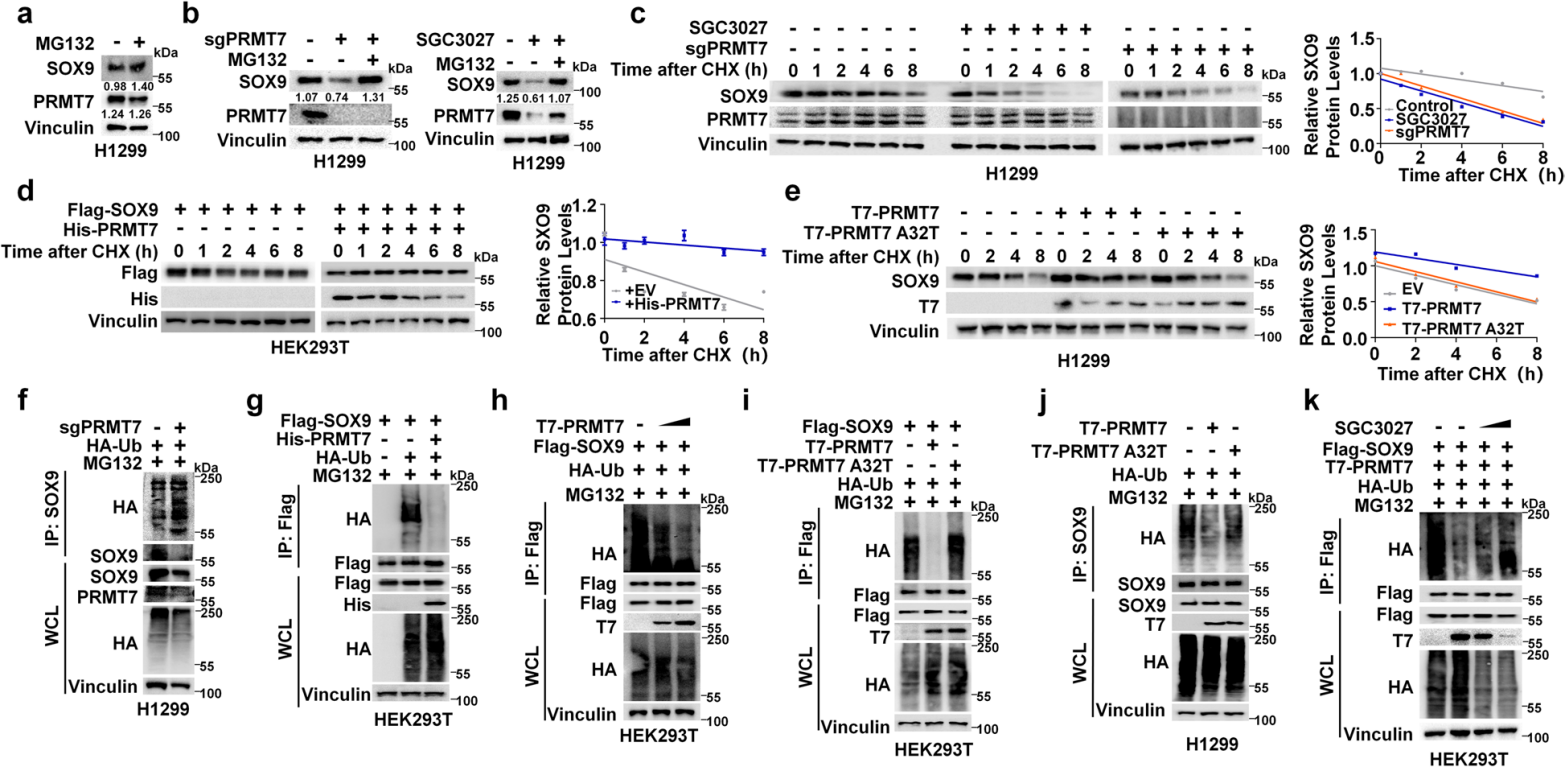
PRMT7 enhanced SOX9 protein stability by inhibiting its ubiquitination and degradation **a** IB analysis of SOX9 and PRMT7 expression in H1299 cells treated with or without 20 μM MG132 for 8 h. **b** IB analysis of SOX9 and PRMT7 expression in cells subjected to either PRMT7 knockout or SGC3027 pretreatment (20 μM, 12 h), followed by treatment with or without MG132 (20 μM) for 8 h. **c** SOX9 protein stability was assessed in H1299 cells following pharmacological inhibition (20 μM SGC3027, 12 h) or genetic knockout of PRMT7. Cells were exposed to 100 μg/mL CHX and collected at specified intervals for immunoblotting (Left). SOX9 band intensities were determined by ImageJ and plotted (Right); data are from three replicates, *n* = 3. **d** SOX9 protein stability was evaluated by cycloheximide (CHX) chase assay. Immunoblots of whole-cell lysates from transfected cells harvested at the indicated time points after treatment with 100 μg/mL CHX (Left). Relative SOX9 band intensities were quantified by ImageJ and plotted (Right), *n* = 3. **e** H1299 cells were transfected for 36 h with T7-PRMT7 or T7-PRMT7 A32T. Cells were treated with 100 μg/mL CHX as indicated times (Left Panel). SOX9 band intensity was quantified by ImageJ software and plotted (Right Panel), *n* = 3. **f** Analysis of SOX9 ubiquitination levels upon PRMT7 knockout. H1299 cells with or without PRMT7 knockout were treated with 20 μM MG132 for 8 h prior to harvest. SOX9 was immunopurified from whole-cell lysates using a specific antibody, and the associated ubiquitin conjugates were detected by immunoblotting with an anti-HA antibody which specifically recognizes HA-tagged ubiquitin. **g** In vivo ubiquitination assay of SOX9 in HEK293T cells with ectopic expression of His-PRMT7. Cells were harvested 36 h post-transfection following an 8-h treatment with 20 μM MG132. Flag-SOX9 was immunopurified from cell lysates using an anti-Flag antibody, and its ubiquitination status was assessed by immunoblotting with an anti-HA antibody to detect HA-ubiquitin conjugates. **h** Dose-dependent effect of PRMT7 on SOX9 ubiquitination in vivo. HEK293T cells were transfected with escalating amounts of T7-PRMT7 plasmid for 36 h, followed by treatment with 20 μM MG132 for 8 h before harvesting. Flag-SOX9 was isolated from cell lysates by immunoprecipitation with an anti-Flag antibody, and ubiquitinated forms were detected by immunoblotting using an anti-HA antibody specific for HA-tagged ubiquitin. **i**, **j** The impact of PRMT7 catalytic activity on SOX9 polyubiquitination. IB analysis of WCL and IP products from HEK293T cells (**i**) or H1299 cells (**j**) transfected with the indicated constructs. Cells were treated with 20 μM MG132 for 8 h before harvesting. **k** To assess the impact of the PRMT7 inhibitor SGC3027 on SOX9 ubiquitination, HEK293T cells transfected with the designated plasmids were subjected to increasing concentrations of SGC3027 (10 μM, 20 μM, 12 h). Subsequently, SOX9 proteins modified by ubiquitin were isolated via immunoprecipitation using an anti-Flag antibody and detected by immunoblotting

Consistent with the well-established degradation of SOX9 via the ubiquitin–proteasome pathway [13–15], treatment with the proteasome inhibitor MG132 increased SOX9 protein levels, whereas PRMT7 levels remained unchanged (Fig. 2a). We hypothesized that SOX9 protein stability is affected through this pathway by PRMT7. Our results indicated that MG132 effectively blocked the decrease in SOX9 protein levels caused by PRMT7 knockout or SGC3027 treatment (Fig. 2b), indicating that PRMT7 increased SOX9 protein levels by inhibiting its ubiquitination. Additionally, overexpression of the WT PRMT7, not the A32T mutant, prolonged the protein half-life of SOX9 (Fig. 2d-e). Furthermore, we observed that depleted PRMT7 increased endogenous SOX9 polyubiquitination, accompanied by a shortened SOX9 protein half-life in H1299 cells (Fig. 2c and f). Importantly, expression of WT PRMT7, but not A32T mutant, significantly reduced SOX9 polyubiquitination in vivo (Fig. 2g-j). Similar to the effect of PRMT7 deficiency, treatment with SGC3027 strongly shortened the SOX9 protein half-life and dose-dependently increased SOX9 polyubiquitination (Fig. 2c and k). Together, these findings demonstrated that PRMT7 maintained the protein stability of SOX9 through inhibiting its ubiquitination and degradation in a methyltransferase activity-dependent manner.

### PRMT7 protects SOX9 from KEAP1- or FBXW7- mediated degradation

**Fig. 3:**
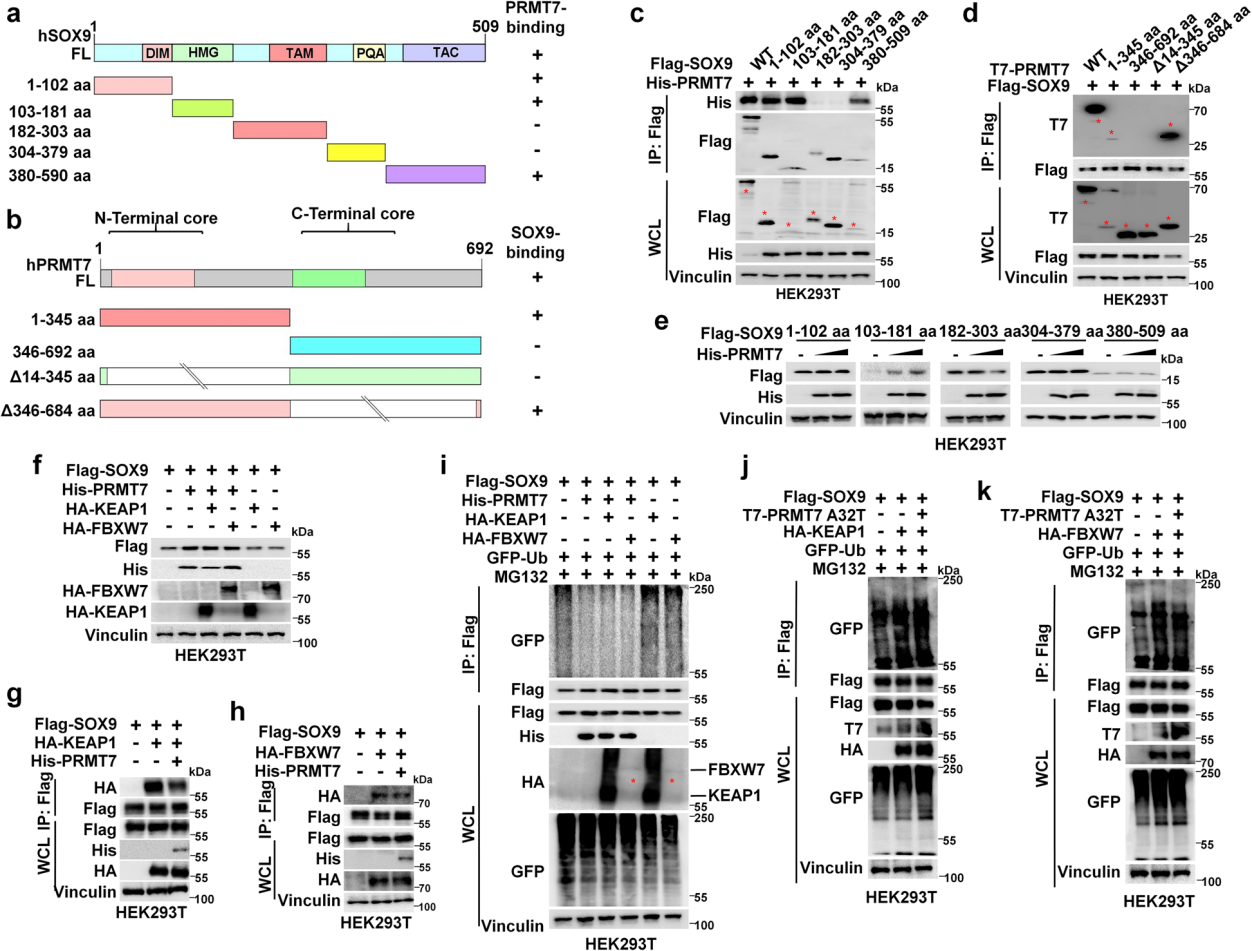
PRMT7 protected SOX9 from KEAP1- or FBXW7-mediated degradation, **a**, **b** Schematic illustration depicting the functional domains of SOX9 (**a**) or PRMT7 (**b**). **c** HEK293T cells were co-transfected with His-PRMT7 and Flag-SOX9 (FL) or an indicated mutant construct for 36 h and then harvested for IP and IB analysis. **d** HEK293T cells were co-transfected with Flag-SOX9 and T7-PRMT7 (FL) or an indicated mutant construct for 36 h and then harvested for IP and IB analysis. **e** The effect of PRMT7 expression on the stability of SOX9 truncation mutants. HEK293T cells were co-transfected with plasmids encoding the specified truncated SOX9 mutants along with increasing amounts of His-tagged PRMT7. Whole-cell lysates were then analyzed by immunoblotting to determine the protein levels of the SOX9 mutants. **f** The protein levels of SOX9 were assessed by IB in HEK293T cells. Cells were co-transfected with Flag-SOX9 along with either HA-KEAP1 or HA-FBXW7, in the presence or absence of His-PRMT7 co-expression, as indicated. **g**, **h** KEAP1 or FBXW7 antagonism of PRMT7-SOX9 interaction. HEK293T cells were co-transfected with Flag-SOX9 and either HA-KEAP1 (**g**) or HA-FBXW7 (**h**), with or without His-PRMT7. After 36 h, cells were harvested, and the lysates were subjected to IB analysis and IP with the indicated antibodies to assess the interaction. **i** In vivo ubiquitination assay was conducted in HEK293T cells co-transfected with Flag-SOX9, His-PRMT7, HA-KEAP1 or HA-FBXW7. SOX9 polyubiquitination was evaluated by IB analysis. Prior to harvesting, cells underwent an 8-h treatment with the proteasome inhibitor MG132 (20 μM). **j**, **k** Investigation of the modulatory role of PRMT7 A32T on SOX9 polyubiquitination in the context of KEAP1 or FBXW7 expression. HEK293T cells were co-transfected with Flag-SOX9, T7-PRMT7 A32T, HA-KEAP1 (**j**) or HA-FBXW7 (**k**). IB analysis of SOX9 polyubiquitination was performed on cells treated for 8 h with 20 μM MG132 to stabilize ubiquitin-conjugated proteins prior to lysis

To elucidate the molecular mechanism by which PRMT7 stabilizes SOX9 protein, we mapped the interaction regions between PRMT7 and SOX9 (Fig. 3a-b). PRMT7 interacts with the DIM, HMG, and TA domains of SOX9 (Fig. 3c), while SOX9 preferentially interacts with the N-terminal domain of PRMT7 (Fig. 3d). Notably, PRMT7 significantly elevates the protein levels of the HMG domain of SOX9 in a dose-dependent manner (Fig. 3e).

We and others have identified that KEAP1 or FBXW7 ubiquitinate and degrade SOX9 protein [13–15], so we investigated whether PRMT7 could antagonize KEAP1- or FBXW7-mediated ubiquitination of SOX9 to maintain its protein stability. Indeed, we observed that overexpression of WT PRMT7, but not A32T mutant, restored the reduced SOX9 protein levels mediated by KEAP1 or FBXW7 (Fig. 3f and Fig. S2a-b). However, we found that overexpression of PRMT7 did not significantly change the KEAP1 or FBXW7 protein levels (Fig. S2c-e), indicating that PRMT7 did not directly decrease KEAP1 or FBXW7 protein levels to maintain SOX9 protein stability. Notably, ectopic expression PRMT7 inhibited KEAP1- or FBXW7 and SOX9 interaction (Fig. 3g-h). Furthermore, overexpression of KEAP1 or FBXW7 increased SOX9 polyubiquitination, but this effect was partially reversed by co-expression of WT PRMT7, not the PRMT7 A32T mutant (Fig. 3i-k). Correspondingly, ectopic expression of PRMT7 failed to further suppress SOX9 ubiquitination upon knockdown of KEAP1 and FBXW7 (Fig S2f). Collectively, these results indicate that PRMT7 antagonizes KEAP1- or FBXW7-mediated SOX9 ubiquitination and degradation, thereby maintaining SOX9 protein stability.

### PRMT7 catalyzed arginine mono-methylation (MMA) of SOX9 at R160 residue

**Fig. 4:**
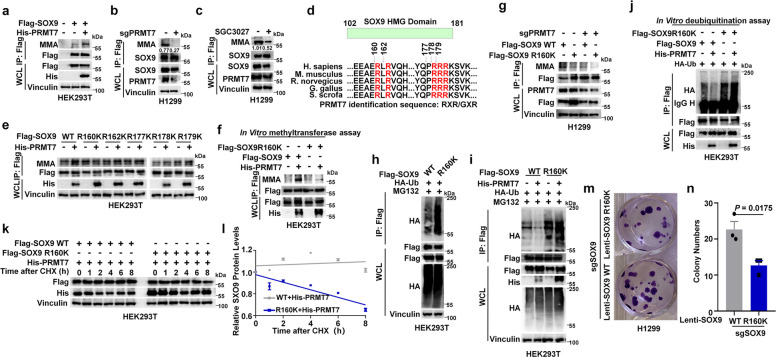
PRMT7 catalyzed arginine mono-methylation of SOX9 at R160 residue. **a** The level of SOX9 mono-methylation is increased in HEK293T cells expressing His-PRMT7. HEK293T cells co-transfected with Flag-SOX9 with or without His-PRMT7. Cell lysates were immunoprecipitated with anti-Flag antibody and anti-MMA antibody was used to detect the mono-methylation level of SOX9. **b** The level of SOX9 mono-methlyation is decreased in PRMT7 KD cells. Lysates from H1299 cells with a stable PRMT7 knockout were immunoprecipitated with anti-SOX9 antibody and anti-MMA antibody was used to detect the mono-methylation level of SOX9. **c** To determine the effect of PRMT7 inhibition on SOX9 mono-methylation, H1299 cells were treated with 20 μM SGC3027 for 12 h. Cell lysates were then subjected to IP using an anti-SOX9 antibody, and the level of mono-methylation on the immunoprecipitated SOX9 was detected by immunoblotting with an anti-mono-methyl arginine (anti-MMA) antibody. **d** A schematic presentation of the evolutionarily conserved putative PRMT7 mono-methylation motif in SOX9. **e** The mono-methylation levels of WT SOX9 and the specified R to K mutants were assessed by IB. HEK293T cells expressing these SOX9 variants were analyzed under conditions of either presence or absence of ectopic His-PRMT7 expression. **f** An in vitro methylation assay was conducted with recombinant Flag-tagged SOX9 and R160K protein purified from HEK293T cells and His-PRMT7 precipitated from HEK293T cells. **g** Effects of PRMT7 knockout by sgRNA in H1299 cells on SOX9-WT and SOX9 R160K mutant MMA level were evaluated by IB analysis. **h** HEK293T cells were transfected with the specified plasmids for an in vivo ubiquitination assay. Subsequently, cells were harvested, and the lysates were subjected to IB analysis to evaluate protein ubiquitination and expression levels. **i** An in vivo ubiquitination assay was performed on SOX9 in HEK293T cells expressing Flag-SOX9 WT or Flag-SOX9 R160K mutant, with or without His-PRMT7 co-expression. Cells were treated with 20 μM MG132 for 8 h before harvesting. **j** In vitro deubiquitination assay using 3 × Flag SOX9-Ub or 3 × Flag SOX9 R160K-Ub as substrate, and His-PRMT7 as enzymes. **k**, **l** IB analysis of SOX9 protein levels in HEK293T cells expressing with His-PRMT7 and Flag-SOX9 WT or Flag-SOX9 R160K mutant followed by treatment with 100 μg/mL CHX at the indicated time points (**k**). Measurement of SOX9 protein levels was performed with ImageJ software, and the quantified data are displayed in the plot (**l**), *n* = 3. **m**, **n** Colony formation assays of H1299 cell lines with stably expressing the indicated SOX9 constructs with endogenous SOX9 knockout by sgRNA. Cell clones were imaged (**m**) and quantified (**n**). Data are expressed as mean ± SEM. *t-*test, *n* = 3

Since PRMT7 methylates β-catenin and inhibits its protein ubiquitination and degradation to maintain its stability [36], we investigated PRMT7 to determine if it affects the methylation of SOX9. Indeed, we observed that the ectopic expression of PRMT7 increased the SOX9 mono-methylation levels (Fig. 4a), whereas PRMT7 knockout or treatment with SGC3027 significantly decreased its mono-methylation levels (Fig. 4b-c), indicating that PRMT7 catalyzed the arginine mono-methylation of SOX9.

Recent studies have demonstrated that PRMT7 recognizes substrates primarily through GAR (Glycine and Arginine) motifs and RXR (Arginine X Arginine) motifs [37, 38]. The R residues of SOX9 proteins with these motifs are predominantly located in the HMG and K2 structural domains. Given that PRMT7 specifically interacts with the HMG domain of SOX9, we examined the conserved R residues within the HMG domain (R160/162/177/178/179), which are conserved across species (Fig. 4d). To identify the specific R residues of SOX9 methylated by PRMT7, we mutated the 5 R residues to lysine (K). In vivo methylation experiments indicated that among the other mutations tested, only the SOX9 R160K mutant reversed the SOX9 methylation levels mediated by ectopic expression of PRMT7 (Fig. 4e). In vitro methylation assays confirmed that PRMT7 directly catalyzes the methylation of WT SOX9, but not the R160K mutant (Fig. 4f). Accordingly, PRMT7 knockout inhibited methylation of WT SOX9 but had little effect on the R160K mutant in cells (Fig. 4g). Compared with SOX9 WT, the SOX9 R160K mutant’s polyubiquitination was increased (Fig. 4h-j), which was accompanied by a shortened SOX9 protein half-life (Fig. 4k-l). In contrast to its effect on WT SOX9, PRMT7 failed to reduce the polyubiquitination of the SOX9 R160K mutant in both in vivo and in vitro deubiquitination assays (Fig. 4i-j). The SOX9 R160K mutant also showed significantly greater inhibition of colony formation in H1299 cells than was observed with the SOX9 WT (Fig. 4m-n). Collectively, these results demonstrated that the PRMT7-mediated mono-methylation of SOX9 at residue R160 stabilized the SOX9 protein and promoted NSCLC proliferation.

### PRMT7 promoted the malignant phenotype of NSCLC depending on SOX9

**Fig. 5:**
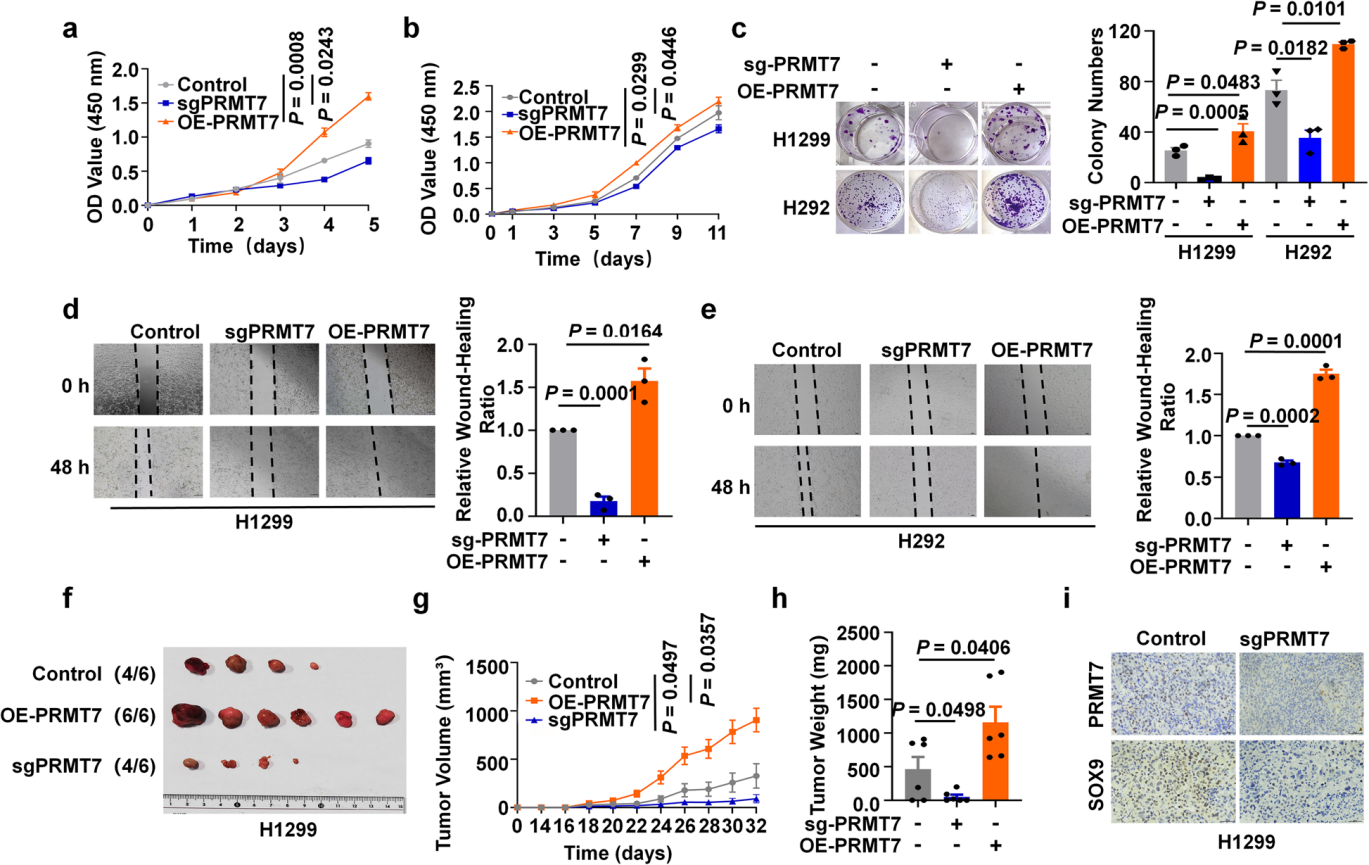
PRMT7 promoted the malignant phenotype of NSCLC. **a**, **b** The proliferation of H1299 cells (**a**) or H292 cells (**b**) transfected with lentivirus for PRMT7 knockout or overexpression was measured by CCK8 assays. per group. *t*-test, *n* = 3. **c** Colony formation assays were conducted on H1299 or H292 cells with PRMT7 depletion, or stable expression of PRMT7. Cell clones were imaged and quantified. Data are expressed as mean ± SEM.* t*-test, *n* = 3. **d**, **e** Wound healing assay conducted on H1299 (**d**) or H292 (**e**) cells with PRMT7 depletion, or stable expression of PRMT7. Data are expressed as mean ± SEM (*n* = 3 per group). *t-*test. **f–h** Stable PRMT7 overexpression or knockout H1299 cells (5 × 10^5^ cells/mice, nude mice), along with control cells were injected subcutaneously into the male BALB/c nude mice. Xenograft growth was measured twice weekly for 32 days (**g**), two-way ANOVA. Xenografts were excised at the endpoint (**f**) and weighed (**h**). Data are expressed as mean ± SEM. *t-*test, *n* = 6. **i** IHC analysis of SOX9 protein level in the PRMT7 knockout cell group from xenograft tumors. Scale bar, 50 μm

A previous study demonstrated that overexpression of PRMT7 promoted invasion and colony formation in NSCLC cell lines in vitro [39]. Our results also revealed its role in enhancing proliferation, migration and colony formation in vitro (Fig. 5a-e). To further investigate the role of PRMT7 in NSCLC progression, we established tumor xenograft models and found that PRMT7 overexpression promoted tumor growth in vivo (Fig. 5f-i). In addition, both genetic knockout of PRMT7 and its pharmacological inhibition by SGC3027 significantly suppressed NSCLC proliferation in vitro and tumorigenesis in vivo (Fig. 5a-i, and S3a-e). IHC analysis of the resulting tumors showed a concomitant decrease in SOX9 protein levels (Fig. 5i), supporting a model wherein PRMT7 functions as an oncogenic driver by sustaining SOX9 protein expression to promote NSCLC progression.

**Fig. 6:**
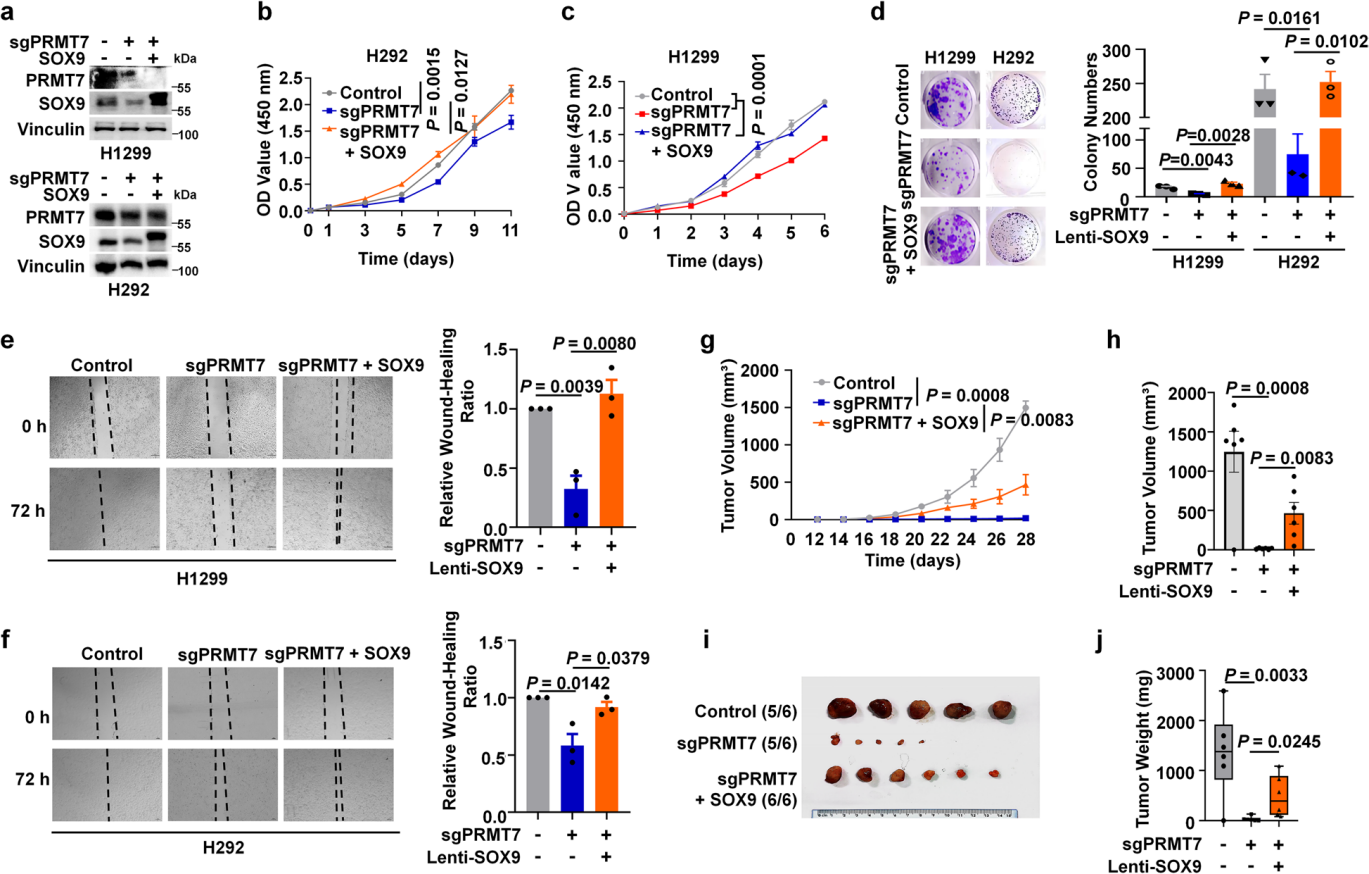
PRMT7 promoted the malignant phenotype of NSCLC relying on SOX9. **a** Stable H1299 or H292 cell lines were generated with PRMT7 knockout and SOX9 overexpression respectively and simultaneously, and the efficacy was validated by IB analysis. **b**, **c** Growth curves of PRMT7-KD H1299 (**c**) and H292 (**b**) cells with or without SOX9 expression were determined by CCK-8 assays. *n* = 3 per group. *t-*test. **d** Colony formation efficiency assays in H1299 or H292 PRMT7-KD cells with or without expressing SOX9 were performed after 14 days. Data are expressed as mean ± SEM (*n* = 3). *t-*test. **e**, **f** Wound healing assay conducted on H1299 (**e**) and H292 (**f**) cells with PRMT7 depletion, either with or without SOX9 overexpression. Data are expressed as mean ± SEM (*n* = 3 per group). *t-*test. **g-j** Xenograft tumor growth of subcutaneous tumors formed by PRMT7-depleted H1299 cells (2.5 × 10^6^ cells/mice, nude mice), either with or without SOX9 overexpression. Tumor growth was monitored at the indicated time points (**g**), two-way ANOVA. Xenograft volume on the final day is shown (**h**). Xenografts were excised at the endpoint (**i**) and weighed (**j**). Data are expressed as mean ± SEM (*n* = 6 per group). *t-*test

To determine whether PRMT7 regulates the malignant phenotype of NSCLC through SOX9, we performed rescue experiments by overexpressing SOX9-WT and SOX9 R160K mutant in PRMT7 deficient cells. In vitro experiments demonstrated that re-expression of SOX9-WT in PRMT7 knockout cells increased their cell proliferation, colony formation, and migration capacity compared R160K mutant (Fig. 6a-f and Fig. S4a-c). Consistently, re-expression of exogenous SOX9-WT rather than SOX9 R160K mutant significantly restored the reduction in tumorigenesis induced by PRMT7 knockout in xenograft mouse models (Fig. 6g-j and S4d-f). Collectively, these findings indicated that PRMT7 promoted the malignant phenotype of NSCLC in vitro and in vivo, at least in part through SOX9.

### High levels of PRMT7 and SOX9 correlated with poor prognosis of NSCLC

**Fig. 7:**
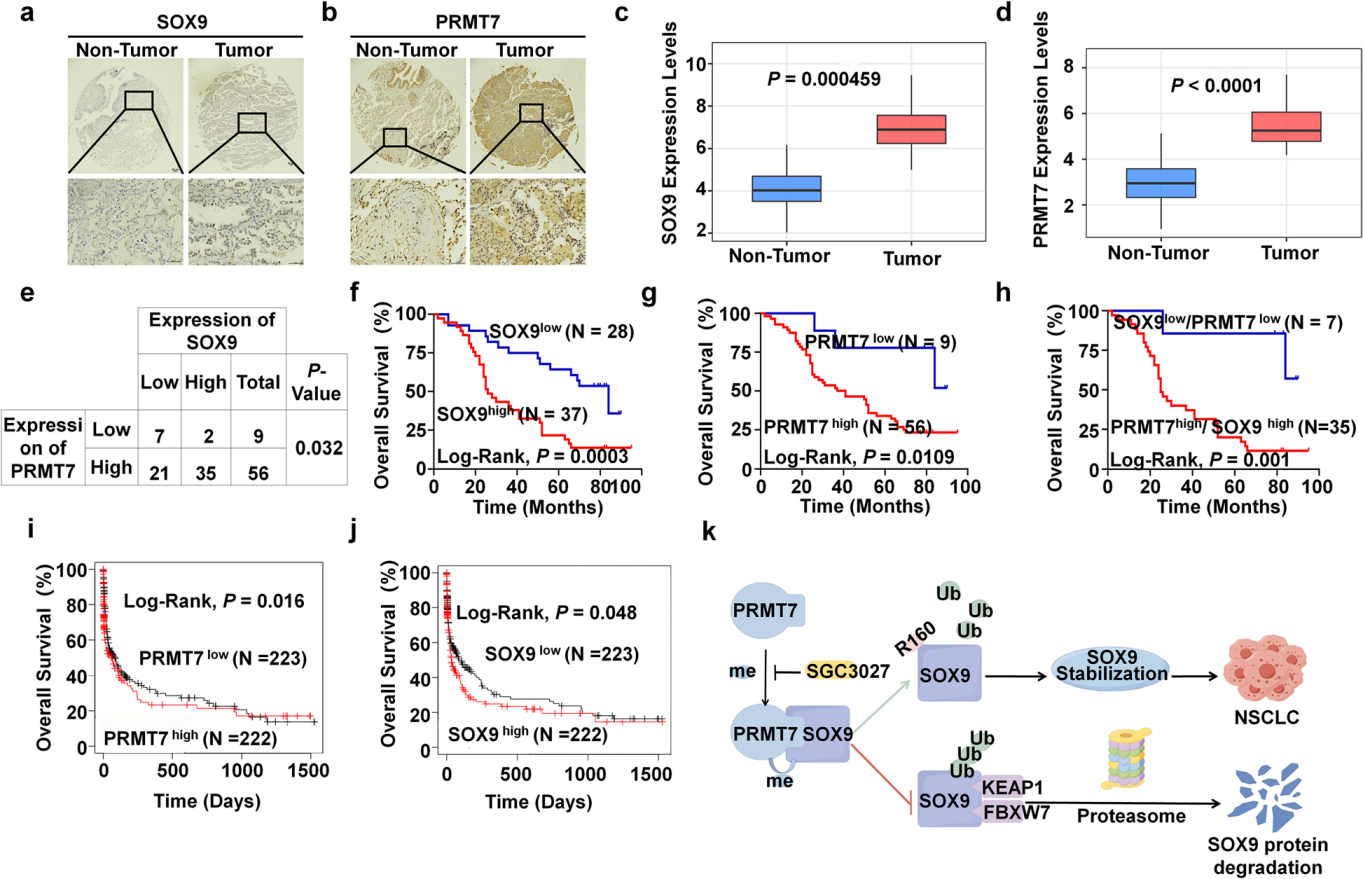
High levels of PRMT7 and SOX9 correlated with poor prognosis of NSCLC. **a**, **b** Representative IHC images indicating SOX9 (**a**) and PRMT7 (**b**) expression in the tissue microarray are shown. Scale bar, 20 μm. **c**, **d** Expression levels of SOX9 (**c**) or PRMT7 (**d**) in lung cancer tissues and normal lung tissues. Processed count data and code used for analyses is available as a Code Ocean capsule from https://doi.org/10.24433/CO.0121060.v1. **e** Correlations between PRMT7 and SOX9 expression in NSCLC patients and statistical analysis of IHC staining. **f**, **g** Kaplan–Meier curves of overall survival for patients with NSCLC stratified by the SOX9 expression level (**f**) or PRM7 expression level (**g**) in the tissue microarray are shown. Data were analyzed using the Log-Rank test. **h** Kaplan–Meier curves of overall survival for patients with NSCLC stratified by SOX9 and PRMT7 expression levels in the tissue microarray are shown. Data were analyzed using the Log-Rank test. **i**, **j** Kaplan–Meier overall survival curves based on PRMT7 and SOX9 expression in lung cancer patients. Data were collected from GEN2 database. (http://gent2.appex.kr/gent2/). **k** A brief working model illustrating that PRMT7-mediated SOX9 arginine mono-methylation at R160 residue antagonizes KEAP1- or FBXW7-mediated SOX9 protein ubiquitination and degradation and promotes NSCLC tumorigenesis. The figure was drawn by Figdraw

Next, we further investigated the clinical relevance of the PRMT7-SOX9 axis in NSCLC. IHC staining and bioinformatics analysis revealed significantly higher expression of PRMT7 and SOX9 in NSCLC tissues than in non-tumor tissues (Fig. 7a-e). Kaplan–Meier survival analysis revealed that the overall survival (OS) of the high levels of SOX9 or PRMT7 was significantly shorter compared with the low levels (Fig. 7f-g). Moreover, patients with high expression of both PRMT7 and SOX9 show a worse prognosis compared to those with low expression of both (Fig. 7h). Similarly, GEN2 datasets indicated that both PRMT7 and SOX9 showed significantly poorer OS for high levels than for lower levels (Fig. 7i-j), which further supported our findings. Collectively, these findings suggest that elevated levels of PRMT7 and SOX9 are associated with a poor prognosis in NSCLC.

## Discussion

Protein arginine methylation, catalyzed by PRMTs, modulates diverse physiological and pathological processes and is increasingly acknowledged as a key contributor to various human diseases, including cancer [23, 25, 28, 40–42]. In the present study, we screened and identified PRMT7 as the specific arginine methyltransferase for SOX9. We demonstrated that PRMT7 methylated SOX9 at R160 residue, thereby antagonizing its ubiquitination and degradation mediated by KEAP1 or FBXW7, and consequently promoting NSCLC proliferation. Furthermore, we established that the PRMT7-SOX9 axis plays a crucial role in NSCLC progression both in vitro and in vivo, highlighting its potential as a promising therapeutic target (Fig. 7k).

SOX9 is a well-characterized oncoprotein that plays a critical role in cell-fate determination, proliferation and differentiation [12, 43]. Its functional diversity is tightly regulated by post-translational modifications (PTMs), which profoundly influence its protein stability and biological activity [13–15, 44–49]. Previous studies have shown that SOX9 can be ubiquitinated by the E3 ligases KEAP1 or FBXW7, leading to its proteasomal degradation and consequent suppression of tumor progression [13–15]. Intriguingly, arginine methylation has emerged as an important regulatory layer in the control of protein stability through the ubiquitin–proteasome system [50–54]. In this study, we demonstrate that PRMT7 interacts with SOX9 and reduces its polyubiquitination in a methyltransferase activity-dependent manner. To elucidate the mechanism, we identified R160 as the specific residue on SOX9 methylated by PRMT7. We further establish that this methylation at R160 directly antagonizes the ubiquitination and degradation of SOX9 mediated by KEAP1 or FBXW7, thereby promoting NSCLC progression. These findings reveal a previously unrecognized crosstalk between arginine methylation and ubiquitination that fine-tunes SOX9 protein homeostasis and tumorigenic activity. Although our findings establish that PRMT7 stabilizes SOX9 primarily by antagonizing its KEAP1/FBXW7-mediated ubiquitination, it remains possible that PRMT7 influences SOX9 stability through additional mechanisms, such as via other E3 ligases or deubiquitinating enzymes. Fully elucidating the broader PRMT7-SOX9 regulatory network will be an important direction for future research.

As the sole enzyme responsible for catalyzing protein MMA modification, PRMT7 contributes to tumorigenesis and cell survival through context-dependent methylation of histone and non-histone substrates [33, 35, 36, 39, 55, 56]. Previous work has suggested that PRMT7 overexpression acts as an oncogene, promoting metastatic traits in NSCLC in vitro [39]. Our study now provides novel mechanistic insights into how PRMT7 drives NSCLC progression. Specifically, we found that re-expression of SOX9 substantially restored the proliferative and tumorigenic capacity of PRMT7-depleted NSCLC cells both in vitro and in vivo, indicating that the oncogenic function of PRMT7 depends, at least in part, on SOX9. In addition to driving tumor growth, it remains unclear whether the PRMT7-SOX9 axis facilitates metastatic dissemination in NSCLC, a question that warrants further elucidation.

Analysis of clinical datasets confirmed that PRMT7 and SOX9 are upregulated in human NSCLC specimens, and their elevated expression was associated with poor patient prognosis, highlighting the translational relevance of this axis. Given that direct targeting of SOX9 has proven difficult, primarily due to its unresolved protein structure, therapeutic inhibition of PRMT7 has emerged as a promising alternative strategy. Nevertheless, the therapeutic potential of this approach is moderated by concerns of on-target toxicity, considering the physiological roles of PRMT7 in processes such as neuronal differentiation [57]. As evidenced by Li et al., a high dose of JS1310 induced considerable toxicity in a prostate cancer model, which motivated the development of a new inhibitor, A33, with enhanced tolerability [58]. Collectively, these findings indicate that dose adjustments or the application of next-generation inhibitors may improve the therapeutic index. Therefore, further in vivo studies and comprehensive long-term safety assessments are imperative to fully evaluate the risk–benefit profile of inhibiting the PRMT7-SOX9 axis in NSCLC.

Our study acknowledges several limitations. First, although we have defined a mechanism through which PRMT7-mediated mono-methylation of SOX9 at R160 blocks its degradation by KEAP1/FBXW7, the involvement of other pathways cannot be excluded, and we are actively investigating other potential E3 ligases that target SOX9. Second, the in vivo analysis relied solely on a single xenograft model; employing complementary models such as PDX or experimental metastasis assays would more comprehensively establish its pathophysiological relevance. Finally, the clinical implications are constrained by the limited sample size, and the translational potential of the PRMT7-SOX9 axis must be validated in larger cohorts.

Overall, we identified PRMT7 as a specific protein arginine methyltransferase for SOX9. Methylation of SOX9 at R160 residue by PRMT7 antagonized KEAP1- or FBXW7-mediated ubiquitination and degradation, stabilizing the SOX9 protein. Consequently, the PRMT7-SOX9 axis drives NSCLC proliferation and tumorigenesis, establishing it as a promising therapeutic target for this malignancy.

## Materials and methods

### Cell culture and transfection

HEK293T and H1299 cells were obtained from Cell Bank, Chinese Academy of Science. H292 was purchased from Pricella Biotechnology (China). All cell lines were cultured in Dulbecco’s Modified Eagle’s Medium (DMEM, Gibco) supplemented with 10% fetal bovine serum (FBS), 100 units/mL penicillin, and 100 µg/mL streptomycin, and maintained at 37 °C in a 5% CO_2_ humidified atmosphere. Cell lines were authenticated by short tandem repeat (STR) profiling and confirmed free of mycoplasma contamination. Upon receipt, cells were expanded, cryopreserved, and used within 3–4 months to ensure viability and genetic stability. Plasmid transfections were performed with Neofect DNA transfection reagent following the manufacturer’s instructions.

### Generation of PRMT7 knockout cell lines using the CRISPR/Cas9 technology

Stable PRMT7 knockout H1299 and H292 cell lines were generated by lentiviral delivery of CRISPR/Cas9. The sgRNA (5’-GTCGGGCCAATCCGACCACG-3’) was cloned into a pLentiCRISPR vector (Chengdu RabbitBio, China). Following transduction, cells were selected with puromycin, and successful knockout was confirmed by immunoblotting.

### Antibodies and chemical reagents

The following antibodies were used in immunoblotting. Anti-T7-Tag (1:1000 dilution, Cat# 13246), anti-HA-Tag (1:1000 dilution, Cat# 3724), anti-GFP-Tag (1:1000 dilution, Cat# 2956), anti-His-Tag (1:1000 dilution, Cat# 12698), Rabbit (DA1E) mAb IgG XP® Isotype Control (Cat# 3900), Mouse (G3A1) mAb IgG1 Isotype Control (Cat# 5415), anti-SOX9 (1:1000 dilution, Cat# 82630), anti-PRMT7 (1:1000 dilution, Cat#14762), MMe-Arginine (1:1000 dilution, Cat# 8015S) and anti-KEAP1 (1:1000 dilution, cat# 8047) were purchased from Cell Signaling Technology. Anti-SOX9 (1:500 dilution, Cat# sc-166505), anti-PRMT7 (1:1000 dilution, Cat# sc-376077), anti-α-Tubulin (1:800 dilution, Cat# sc-23948) were purchased from Santa Cruz Biotechnology. Anti-Vinculin (1:1000 dilution, Cat# V9131) and anti-Flag-Tag (1:1000 dilution, Cat# F1804) were purchased from Sigma-Aldrich. Anti-Lamin B1 (1:1000 dilution, Cat# ET1606-27) was purchased from HuaBio. MG132 (Cat# S2619) was purchased from Selleck Chemicals. Anti-FBXW7 (1:1000 dilution, Cat# ab109617) was purchased from Abcam. Cycloheximide (CHX) (Cat# N11534) was purchased from Sigma-Aldrich. SGC3027 (Cat# HY-11245) was purchased from MedChemExpress.

### Plasmids and shRNAs

Expression vectors containing His-tagged PRMTs constructs (PRMT 1, 2, 3, 4, 5, 6, 7, 8, and 9) were purchased from Sino Biological. HA-KEAP1, HA-FBXW7, Flag-SOX9, Flag-SOX9 (1–102 aa), Flag-SOX9 (103–181 aa), Flag-SOX9 (182–303 aa), Flag-SOX9 (304–379 aa) and Flag-SOX9 (380–509 aa) were described previously [15]. GFP-Ubiquitin (Ub) and HA-Ub were purchased from Addgene. Flag-SOX9 R160K, Flag-SOX9 R162K, Flag-SOX9 R177K, Flag-SOX9 R178K, Flag-SOX9 R179K, T7-PRMT7, T7-PRMT7 A32T, T7-PRMT7 (1–345 aa), T7-PRMT7 (346–692 aa), T7-PRMT7 (Δ14–345 aa), T7-PRMT7 (Δ346–684 aa) were generated by subcloning the corresponding cDNAs into the pcDNA3.1 vector. The shRNA oligonucleotide sequences were: non-targeting (NT) shRNA, 5’-CGTACGCGGAATACTTCGA-3’, shKEAP1, 5’-GCACTGCAAATAACCCATCTT-3’; shFBXW7, 5’-CCAGTCGTTAACAAGTGGAAT-3’.

### RNA extraction and Quantitative real-time PCR (qRT-PCR) analysis

Total RNA was isolated from cells using Trizol reagent (TakaRa, #9109). Subsequently, 1 µg of the extracted RNA was reverse-transcribed into cDNA using the PrimeScript RT reagent Kit with gDNA Eraser (TakaRa, #RR092A). qRT-PCR was then performed with SYBR Select Master Mix (TakaRa, #RR820A) on a Bio-Red CFX Manager 3.1 system. Gene expression was normalized to GAPDH, and results are presented as the mean ± SEM from at least three independent experiments. The primer sequences used were: hPRMT7 Forward: 5’-GCTGGAGGAGGATGAACACTATG-3’, Reverse: 5’-CAGCCCGGATACCTTGGTAGTA-3’; hSOX9: Forward: 5’-GACTTCTGAACGAGAGCGAGA-3’, Reverse: 5’-CCGTTCTTCACCGACTTCCTC-3’; hGAPDH Forward: 5’-TGGTATCGTGGAAGGACTC-3’, Reverse: 5’- AGTAGAGGCAGGGATGATG-3’.

### Immunoblotting (IB) and immunoprecipitation (IP) analyses

For immunoblotting (IB) and immunoprecipitation (IP), cells were lysed in ice-cold IP Lysis Buffer (Thermo Fisher Scientific, Cat# 87788) supplemented with protease and phosphatase inhibitors (Roche, Cat# A32965, A32957). After 30 min on ice, lysates were centrifuged (12,000 rpm, 5 min, 4 °C) to remove debris. Protein samples were denatured in 4 × loading buffer (10 min, 100 °C), separated by SDS-PAGE, and transferred to nitrocellulose membranes (Millipore). Membranes were blocked with 5% milk (2 h, RT) and incubated overnight at 4 °C with primary antibodies, followed by HRP-conjugated secondary antibodies. Signals were detected using ECL substrate (Thermo Fisher Scientific, Cat# 34096) and quantified with ImageJ. For IP, cell lysates were incubated with indicated antibodies (2–3 µg) overnight at 4 °C, followed by a 6-h incubation with Protein A/G Sepharose beads (Thermo Fisher Scientific, Cat# 78610). The beads were then washed with IP lysis buffer, and the immunoprecipitated complexes were resuspended in 4 × loading buffer for IB analysis.

### Subcellular fractionation assays

Nuclear and cytoplasmic protein fractions were isolated using a commercial subcellular fractionation kit (Thermo Fisher Scientific, Cat# 78833) according to the manufacturer’s instructions. Following extraction, IB analysis was performed on the isolated nuclear and cytoplasmic protein fractions.

### Protein half-life assay

HEK293T and H1299 cells were transfected with the indicated constructs. Following a 36 h incubation period, 100 µg/mL CHX was added to the cells. Cell samples were collected at designated time intervals, and SOX9 protein stability was subsequently assessed by IB analysis.

### In vivo ubiquitination assay

In vivo ubiquitination assays were carried out following a published method [36]. HEK293T and H1299 cells, after 36-h transfection with designated plasmids, were exposed to 20 µM MG132 for 8 h. Subsequently, cells were harvested and processed as follows: lysis and homogenization; centrifugation (15,000 × g, 4 ℃, 10 min); overnight immunoprecipitation of the supernatant with target antibody at 4 °C; pull-down of complexes with Protein A/G beads (6 h, 4 °C); three washes with IP buffer; and final analysis of the eluates by SDS-PAGE and immunoblotting with anti-tag antibodies.

### In vitro deubiquitination assay

HEK293T cells were transfected together with the Flag-SOX9 or Flag-SOX9 R160K and HA-Ub expression plasmids for 48 h. The cells were treated with MG132 for 6 h. Flag-SOX9 protein was purified by Flag-tag Protein IP Assay Kit with Magnetic Beads (Beyotime P2181M). Separately, His-PRMT7 was expressed in HEK293T cells and purified using nickel-nitrilotriacetic acid (Ni–NTA) matrices (QIAGEN, 31314). The ubiquitinated Flag-SOX9 was then incubated with recombinant His-PRMT7 in deubiquitination reaction buffer (10 mM DTT, 1 mM EDTA, 50 mM Tris–HCl (pH 8.0), 50 mM NaCl, 5% glycerol) at 37 °C for 30 min. The reaction products were analyzed by immunoblotting to evaluate changes in SOX9 ubiquitination levels.

### In vitro methyltransferase assay

HEK293T cells were transfected with plasmids encoding Flag-SOX9 (wild-type or R160K mutant), either alone or together with His-PRMT7. Flag-SOX9 was immunopurified using anti-Flag magnetic beads (Beyotime, P2181M). The immunopurified complexes were incubated in a 20 µL reaction buffer (20 mM Tris pH 8.0, 50 mM KCl, 1 mM DTT, 1 mM PMSF) containing 250 µM S-adenosylmethionine (SAM; Sigma-Aldrich, A4377) at 30 °C for 90 min. The reactions were stopped by adding SDS loading buffer and boiling, and the methylation levels were assessed by immunoblotting.

### Immunofluorescence (IF) analysis

Immunofluorescence staining was performed as previously described [36]. In brief, cells were fixed with 4% paraformaldehyde for 30 min and permeabilized with 0.25% Triton X-100 for 7 min. Subsequently, cells were blocked with 10% FBS and incubated with primary and fluorescently-conjugated secondary antibodies, along with 4’,6-diamidino-2-phenylindole (DAPI, Sigma-Aldrich, Cat# D9542) for nuclear staining. Finally, the glycerol seal was dropped on the slide and observed with Leica confocal microscope.

### Colony formation assay

H1299 and H292 cells were seeded at low density (500–800 cells/well in 12-well plates) and cultured until visible colonies appeared. The colonies were then fixed with methanol for 30 min, stained with 0.4% crystal violet for 15 min, and gently washed. After air-drying, the colonies were counted and quantified using ImageJ software to determine the colony formation rate.

### Cell viability assay

Cell proliferation was measured by CCK-8 assay. Cells were seeded in 96-well plates at 1,000 cells per well. After incubation, 10% CCK-8 reagent was added. Following 2 h incubation, the absorbance at 450 nm was measured to quantify viability.

### Wound healing assay

Briefly, a standardized wound was introduced into a confluent cell monolayer in 6-well plates using a sterile tip. After washing and replacing the medium with serum-free conditions, wound closure was documented by imaging at the indicated time points.

### Immunohistochemistry (IHC) of NSCLC tissue microarray

Human NSCLC tissue microarrays (formalin-fixed, paraffin-embedded) were commercially obtained from Shanghai Outdo Biotech (HLugA180Su11; Approval No. SHYJS-CP-2206001). IHC staining was conducted using a standard kit (ZSGB-Bio, Beijing, China) following the manufacturer’s protocol and a published method [59]. Tissue sections were stained with anti-SOX9 (Cell Signaling Technology, Cat# 82,630; 1:400) and anti-PRMT7 (Thermo Fisher Scientific, PA5-30,743; 1:800) antibodies. The staining intensity of PRMT7 and SOX9 was quantified as previously described [59]. The final IHC score (ranging from 0 to 12) was calculated by multiplying the percentage of positive cells (scored 0–4) by the staining intensity (scored 0–3).

### In vivo xenograft assay

H1299 cells (5 × 10^5^–5 × 10^6^) from the control, OE-PRMT7, sgPRMT7, and sgPRMT7 + SOX9 and sgPRMT7 + SOX9 R160K groups were subcutaneously injected into flanks of randomly assigned 6–8 week old male BALB/c nude mice or 6–8 week old male NOD/SCID mice. Tumor size was measured every 2–3 days with a caliper, and volume was computed as (L × W^2^ × π/6). Upon completion of the study, mice were euthanized, tumors were excised and their weight and volume were quantified. All procedures were conducted in the specific pathogen-free (SPF) facility of the Second Affiliated Hospital of Chongqing Medical University and were approved by the Ethics of the Second Affiliated Hospital of Chongqing Medical University (Approval No. IACUC-SAHCQMU-2024–00180).

### Statistical analysis

Data analysis was performed with GraphPad Prism 10.8 software. Group comparisons for results and growth curves of tumor volumes were evaluated by two-tailed unpaired or paired Student’s *t*-test or two-way ANOVA, as applicable. The correlation between variables was assessed via Pearson correlation analysis. The Log-Rank test was utilized to evaluate the statistical significance of Kaplan–Meier survival curves. Experiments were independently repeated at least three times. Data are presented as mean ± SEM, and a *P*-value < 0.05 was defined as statistically significant.

## Supplementary Information

Supplementary Material 1.

## Data Availability

Not applicable.
