# Supplementary material for: Protein arginine methyltransferase 7-mediated arginine mono-methylation stabilizes SRY-box transcription factor 9 to promote non-small cell lung cancer progression
Source: Mol Biomed. 2025 Dec 22;6:148. doi: 10.1186/s43556-025-00378-0 (PMC12722591; doi:10.1186/s43556-025-00378-0)
Supplement: Supplementary file 1 — Supplementary Material 1. [file 43556_2025_378_MOESM1_ESM.docx]

**Protein arginine methyltransferase 7-mediated arginine mono-methylation stabilizes SRY‑box**

**transcription factor 9 to promote non-small cell lung cancer progression**

Lin Zhang^1^**^#^**, Jingyi Xiang^1^**^#^**, Yali Feng^1, 2^, Gufang Shen^1^, Xu Huang^1, 3^, Tianshu Fang^1^, Yunjia Zhu^1,4^, Hong Ren^1^, Chungang Liu^1*^

^1^Key Laboratory of Molecular Biology for Infectious Diseases (Ministry of Education), Institute for Viral Hepatitis, Department of Infectious Diseases, The Second Affiliated Hospital, Chongqing Medical University, Chongqing 400010, PR China.

^2^Mental Health Center of Jiulongpo District, Chongqing 401329, PR China.

^3^Department of Epidemiology, The First Affiliated Hospital of Shandong First Medical University & Shandong Provincial Qianfoshan Hospital, Jinan, Shandong Province 250000, PR China.

^4^Department of Pathology, The Second Affiliated Hospital of Chongqing Medical University, Chongqing 400010, PR China.

^#^These authors contributed equally.

*Correspondence: C. L.: [liuchungang@zju.edu.cn](mailto:liuchungang@zju.edu.cn) or [liuchungang@hospital.cqmu.edu.cn](mailto:liuchungang@hospital.cqmu.edu.cn).

**Supplementary figures and figure legends**


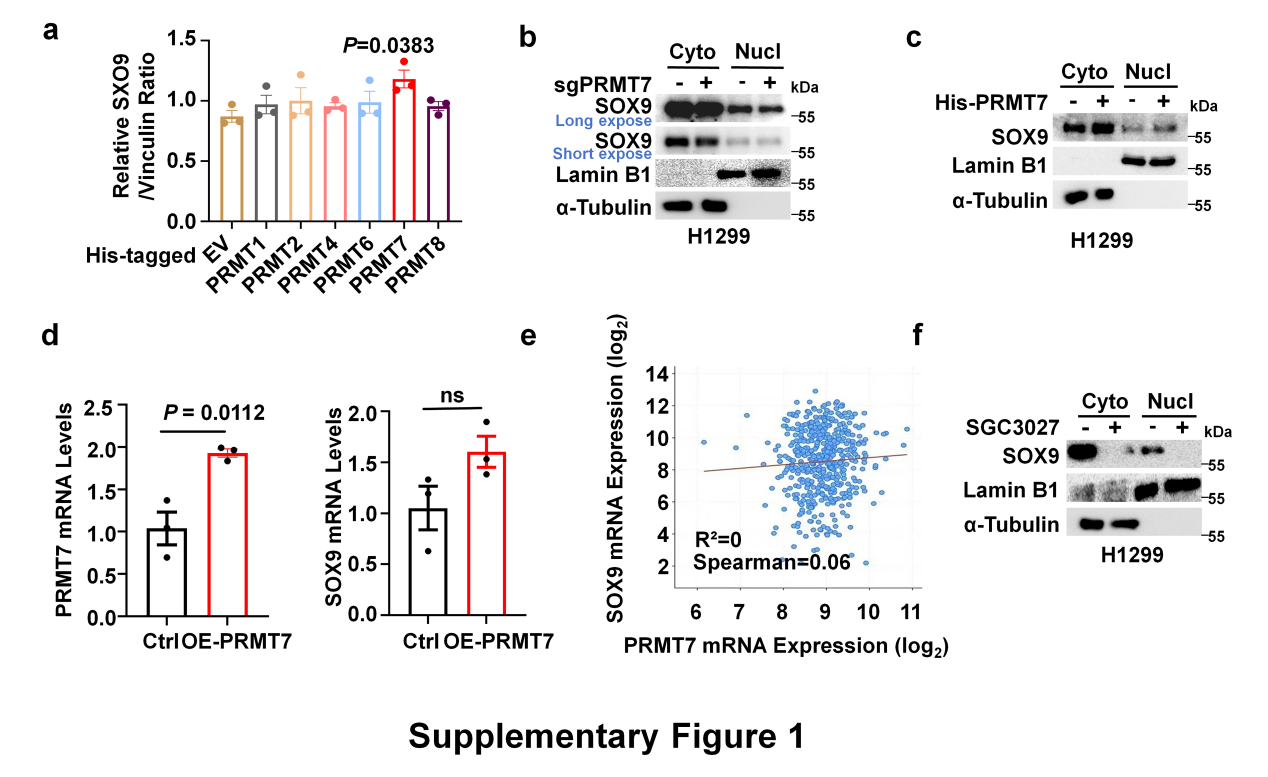


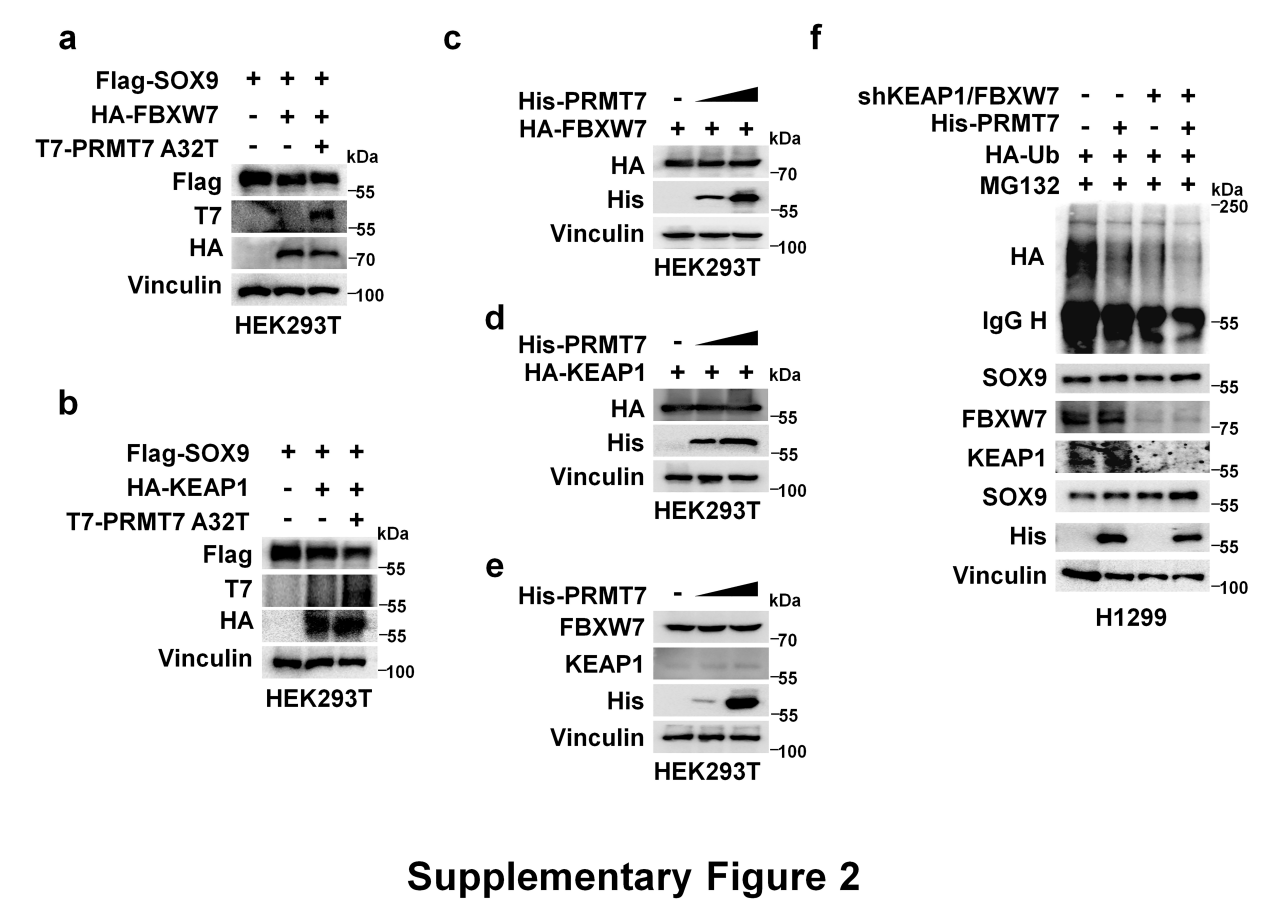


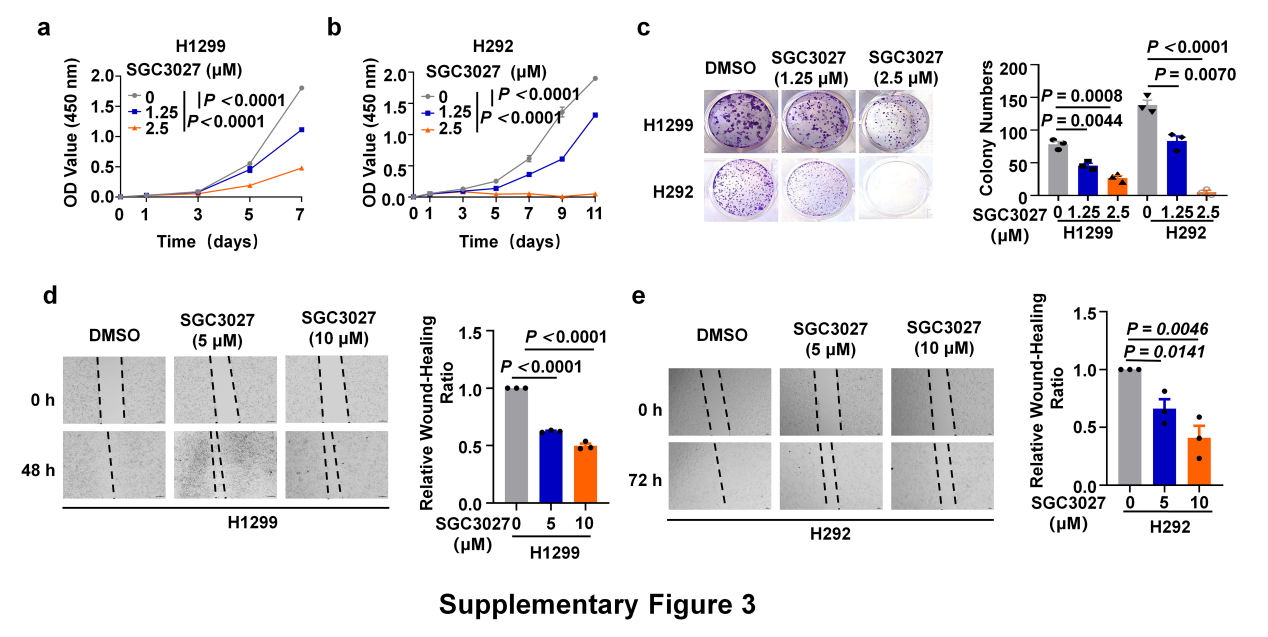


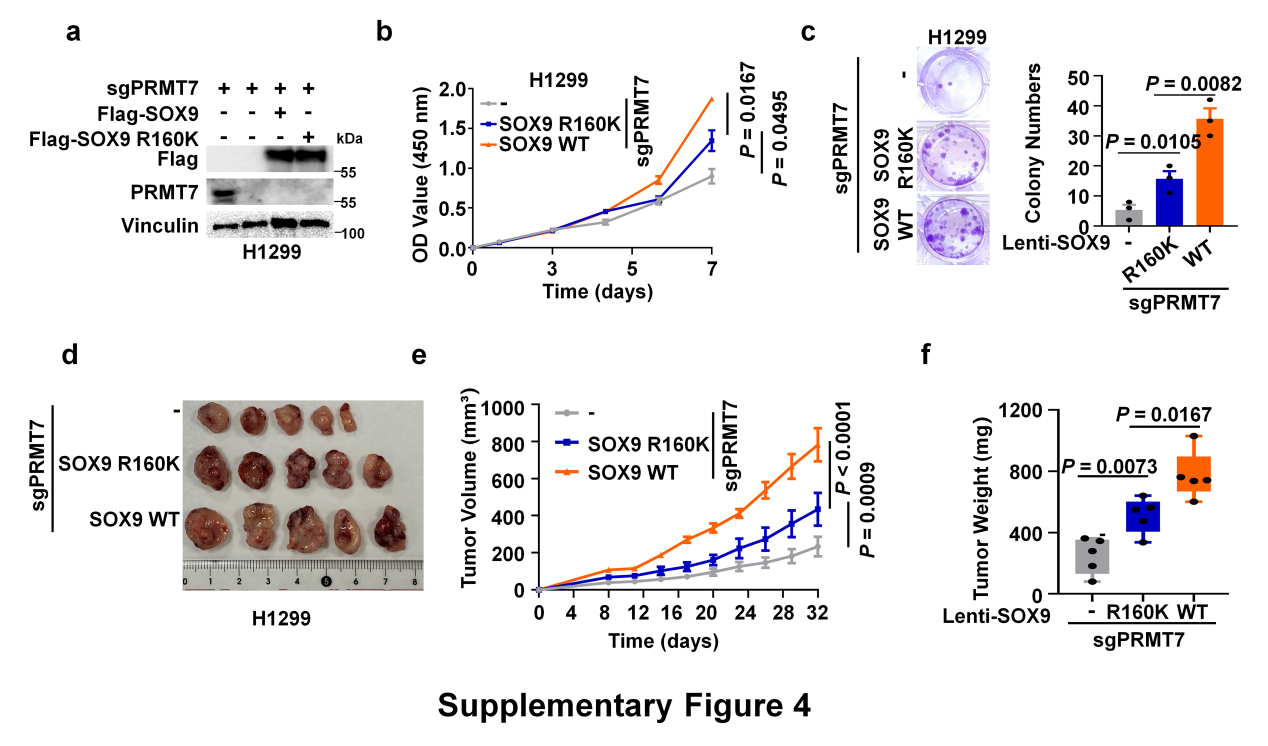


**Supplementary Figure Legends**

**Fig. S1 PRMT7 positively regulates SOX9 protein levels**

**a** Quantification of the SOX9 protein abundance in Figure 1b by the ImageJ software. n = 3 per group. * *P* < 0.05. *t-*test.

**b** Nuclear and cytoplasmic fractions were prepared from H1299 cells transfected with or without sgPRMT7. Nuclear Lamin B1 and cytoplasmic α-Tubulin were used as controls.

**c** Nuclear and cytoplasmic fractions were prepared from H1299 cells expression with or without His-PRMT7. Nuclear Lamin B1 and cytoplasmic α-Tubulin were used as controls.

**d** QRT-PCR analysis of PRMT7 or SOX9 mRNA levels in H1299 cells tranfected with EV or His-PRMT7. n = 3 per group. ns: no significance, * *P* < 0.05, *t-*test.

**e** SOX9 mRNA levels do not correlates with PRMT7 mRNA levels from the lung carcinoma datasets of cBioPortal.

**f** Nuclear and cytoplasmic fractions were prepared from H1299 cells treated with or without 20 μM SGC3027 for 12 hours before harvesting. Nuclear Lamin B1 and cytoplasmic α-Tubulin were used as controls.

**Fig. S2 PRMT7 protected SOX9 from KEAP1- or FBXW7- mediated degradation**

**a** IB analysis of SOX9 protein levels in HEK293T cells co-expressing Flag-SOX9 and HA-FBXW7, with or without T7-PRMT7 A32T.

**b** IB analysis of SOX9 protein levels in HEK293T cells co-expressing Flag-SOX9 and HA-KEAP1, with or without T7-PRMT7 A32T.

**c** IB analysis of FBXW7 protein levels in HEK293T cells expressing increasing amounts of His-PRMT7.

**d** IB analysis of KEAP1 protein levels in HEK293T cells expressing increasing amounts of His-PRMT7.

**e** IB analysis of endogenous FBXW7 or KEAP1 protein levels in H1299 cells transfected with increasing amounts of His-PRMT7.

**f** Effects of PRMT7 expression in H1299 sgKEAP1/sgFBXW7cells on SOX9 ubiquitination were evaluated by IB analysis. Cells were treated with 20 μM MG132 for 8 hours before harvesting. SOX9 was immunoprecipitated with anti-SOX9 antibody and immunoblotted with anti-HA antibody which specifically recognizes HA-tagged ubiquitin.

**Fig. S3 PRMT7 inhibit or SGC3027 inhibited NSCLC cell proliferation and migration**

**a, b** Cell viability of H1299 cells (**a**) or H292 cells (**b**) after treatment with increasing concentrations of SGC3027, conducted by CCK8 assays. n = 3 per group. **** *P* < 0.0001. *t-*test.

**c** Clonogenic survival of H1299 or H292 cells after treatment with increasing concentrations of SGC3027, as assessed by colony-formation assays. Cell clones were imaged and quantified. Data are expressed as mean ± SEM (n = 3 per group). ** *P* < 0.01, *** *P* < 0.001. *t-*test.

**d, e** Wound healing assay conducted on H1299 (**d**) or H292 (**e**) cells after treatment with increasing concentrations of SGC3027. Data are expressed as mean ± SEM (n = 3 per group). * *P* < 0.05, *** *P* < 0.001. *t-*test.

**Fig. S4 PRMT7 promoted the malignant phenotype of NSCLC relying on SOX9 WT rather than R160K mutant**

**a** Stable H1299 cell lines were generated with PRMT7 knockout and Flag-SOX9 or Flag-SOX9 R160K overexpression respectively and simultaneously, and the efficacy was validated by IB analysis.

**b** Growth curves of PRMT7-KD H1299 cells with or without SOX9/SOX9 R160K mutant expression were determined by CCK-8 assays. n = 3 per group. *t*-test .

**c** Clone formation efficiency assays in H1299 PRMT7-KD cells with or without expressing SOX9/SOX9 R160K were performed after 12 days. Data are expressed as mean ± SEM (n = 3). *t*-test.

**d-f** Xenograft tumor growth of subcutaneous tumors formed by PRMT7-depleted H1299 cells, either with or without SOX9/R160K overexpression (8×10^6^ cells/mice, nod/scid mice). Tumor growth was monitored at the indicated time points (**e**), two-way ANOVA. Xenografts were excised at the endpoint (**d**) and weighed (**f**). Data are expressed as mean ± SEM (n=5 per group). *t*-test.
